# Assessment of the Physical, Mechanical and Acoustic Properties of *Arundo donax* L. Biomass in Low Pressure and Temperature Particleboards

**DOI:** 10.3390/polym12061361

**Published:** 2020-06-17

**Authors:** Maria Teresa Ferrandez-García, Antonio Ferrandez-Garcia, Teresa Garcia-Ortuño, Clara Eugenia Ferrandez-Garcia, Manuel Ferrandez-Villena

**Affiliations:** Department of Engineering, Universidad Miguel Hernandez, 03300 Orihuela, Spain; mt.ferrandez@umh.es (M.T.F.-G.); antonio.ferrandezg@umh.es (A.F.-G.); tgarcia@umh.es (T.G.-O.); cferrandez@umh.es (C.E.F.-G.)

**Keywords:** giant reed, composite, physical, mechanical and acoustic properties

## Abstract

Traditionally, plant fibres have been used as a raw material for manufacturing construction materials; however, in the last century, they have been replaced by new mineral and synthetic materials with manufacturing processes that consume a large amount of energy. The objective of this study was to determine the mechanical, physical and acoustic properties of panels made from giant reed residues. The article focuses on evaluating the acoustic absorption of the boards for use in buildings. The materials used were reed particles and urea–formaldehyde was used as an adhesive. The panels were produced with three particle sizes and the influence that this parameter had on the properties of the board was evaluated. To determine the absorption coefficient, samples were tested at frequencies ranging from 50 to 6300 Hz. The results showed that the boards had a medium absorption coefficient for the low and high frequency range, with significant differences depending on the particle size. The boards with 2–4 mm particles could be classified as Class D sound absorbers, while boards with particle sizes of 0.25–1 mm showed the greatest sound transmission loss. Unlike the acoustic properties, the smaller the particle size used, the better the mechanical properties of the boards. The results showed that this may be an appropriate sound insulation material for commercial use.

## 1. Introduction

A material is considered to be suitable for acoustic insulation when it is capable of absorbing sound and has a high sound absorption coefficient in all or part of the spectrum of sound frequencies that are audible to the human ear, ranging from 20 Hz to 20 kHz. These days, there are technical materials on the market that offer good acoustic properties, including those of mineral origin (glass wool, rock wool, etc.) and those of synthetic origin (foam polyurethane, polystyrene, etc.), which not only have a high energy consumption during their manufacture, but also have the disadvantage of not being biodegradable. Due to the environmental problems generated by their manufacture, the use of renewable and ecological resources is increasing, so the use of sustainable materials is becoming a common practice for reducing noise in buildings. One of the plant materials used as a sound absorber is wood in the form of panels or particleboards. The wood residues used to produce wood-based materials have been considered environmentally sustainable, economically viable and socially acceptable [1], but we are seeking suitable replacements to avoid deforestation. Attempts have been made to reduce the use of wood in particleboards by introducing mixtures of forestry waste [2], whole trees [3], prunings from garden trees [4,5] and prunings from fruit trees [6]. In order to meet the demand caused by the decline in supplies of solid wood and wood-based materials, there has been a considerable increase in research into the use of plant fibres for the development of new acoustic insulating products.

In general, plant materials are porous and are good sound absorbers, with good acoustic insulating properties throughout a wide range of frequencies. The fact that plant fibres can be cheaper, lighter and more environmentally friendly explains why they have been studied as an alternative to synthetic fibres [7]. Some plant fibres, such as kenaf, are already on the market, so they could start to be used more extensively. Asdrubali et al. [8] state that the performance of plant fibres is similar to that of synthetic fibres. As a replacement for acoustic materials, various studies have been carried out with particleboards using different types of plant waste [9,10,11,12,13,14,15], including rice stalks [15,16], coconut [17,18,19,20,21,22,23], kenaf [24,25,26], bamboo [27,28,29], sisal [18,26,30], luffa [31], sugarcane [32], jute with latex [33], oil palm [34,35,36,37,38,39], *Arenga pinnata* [40,41], date palm [23,42] and Washingtonia palm [43].

Yang et al. [15] manufactured acoustic panels from rice stalks, with a specific density of 400 and 600 kg·m^−3^, which were suitable as an insulating material for sound absorption in wooden constructions. This study showed that, regardless of the particle size that was used, there was a decrease in the sound absorption coefficient for medium frequencies and an increase in sound absorption in the low- and high-frequency range. Other works using date palm [23], bamboo [29] and oil palm [39] obtained panels that offered good sound absorbing properties at high and low frequencies, and with very low values at medium frequencies. With coconut fibres panels [19], it was found that increasing the thickness improved the acoustic properties, while with low-density palm rachis fibre panels (77 kg·m^−3^, 100 kg·m^−3^ and 125 kg·m^−3^), Khidir et al. [42] showed that the sound absorption coefficient could be increased by increasing the density.

Giant reed (Arundo donax L.) was used as a building material in many Mediterranean countries. In the south of the province of Alicante it was used in all buildings up to the beginning of the 20th century, mainly forming part of the roof and floor. It continued to be used in small detached houses and farm buildings until the 1960s. However, it is no longer used, causing it to spread along riverbanks at a great rate, invading watercourses, impoverishing riverside vegetation and blocking the infrastructures that cross watercourses and making drainage difficult. When the water level rises more than usual, this leads to flooding that causes environmental, economic and material damage. These problems mean that the competent authorities have to make significant economic investments in cleaning and clearing this plant waste from riverbanks and subsequently processing it in authorised landfills. Experiences in Greece [44], Italy [45] and the United Kingdom [46] obtained dry matter yields of Arundo donax L. of 15 t/ha in sterile soils and as much as 40 t/ha in fertile soils, so it is a plant from which a large amount of dry matter can be generated.

The chemical composition of giant reed (Table 1) shows slight variations between the nodes and internodes of the plant and between different authors. It can be concluded that giant reed has a high holocellulose content and a lower lignin content [47,48,49]. The analysis by Shalatov et al. [47] also indicates that it has a high silicate content.

The objective of this study was to evaluate the influence of particle size on the properties of boards manufactured with a process that uses a lower temperature and pressure than commercial boards and to determine whether they can be used as a construction material. The use of giant reed would contribute to the control and recycling of this waste material and would lead to greater energy efficiency and less environmental pollution.

## 2. Materials and Methods 

### 2.1. Materials

The material used was giant reed biomass collected during the clearing of the banks of the River Segura, in southeastern Spain. The reeds were left to dry outdoors for 6 months. They were then shredded in a blade mill. The particles obtained were sorted in a vibrating sieve and three particle sizes were selected: particles that passed through the 4-mm sieve but were retained in the 2-mm one (2 to 4 mm), particles that passed through the 2-mm sieve but were retained in the 1-mm one (1 to 2 mm) and particles that passed through the 1-mm sieve but were retained in the 0.25-mm one (0.25 to 1 mm). The approximate moisture content of the particles was 11%.

Based on previous tests [50], 8 wt % (based on the weight of the particles) of class E1 urea–formaldehyde (UF) with a solid content concentration of 65% and a reaction time of 3–4 h was used as a binder. Ammonium nitrate, at a concentration of 0.4 wt % (based on the weight of the particles), was used as a hardener. The binder was diluted with 2 wt % of water (based on the weight of the binder). No paraffin or water-repellent substance was used.

### 2.2. Manufacturing Process

Three types of panels with different particle sizes (0.25 to 1 mm, 1 to 2 mm and 2 to 4 mm) were produced from the reeds. The UF resin was mixed into the particles in an Imal horizontal axis mixer (model LGB100, Modena, Italy) at 30 rpm for 5 min.

**Figure 1:**
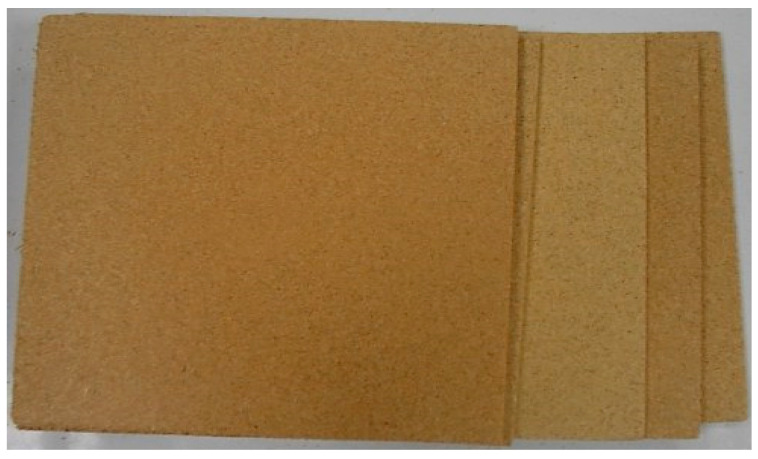
Type A giant reed boards manufactured.

To make the agglomerated particleboards, the mat was formed in a mould of dimensions 400 mm × 600 mm and was subjected to a pressure of 2.6 MPa and a temperature of 120 °C for 4 min in a hot plate press. The formed boards were left to cool outdoors in a vertical position for 24 h. The production characteristics of the three types of panels are shown in Table 2 and several of the panels are shown in Figure 1. Eight boards of each type were manufactured. The particleboards consisted of a single layer and their approximate dimensions were 600 mm × 400 mm × 7.5 mm.

### 2.3. Methods

The method followed to evaluate the particleboards was experimental, conducting tests in the materials strength laboratory of the Higher Technical College of Orihuela at Universidad Miguel Hernández, Elche. Scanning electron microscopy (SEM) was used to determine the texture and analyse the morphological properties of the giant reed. Micrographs were taken of the reed and of cross-sections of the three types of boards. A Hitachi S3000N electron microscope (Hitachi, Ltd., Tokyo, Japan) was used.

Their properties were determined according to the European standards established for wood particleboards [51]. The properties of the boards were measured according to the European standards: density [52], internal bonding strength (IB) [53], modulus of elasticity (MOE) and modulus of rupture (MOR) [54] and thickness swelling (TS) after 2 and 24 h immersed in water [55].

The mechanical tests were performed with a universal testing machine (model IB600, Imal, S.R.L., Modena, Italy). The bending test was carried out with six samples from each board (three in a longitudinal direction and three in a transversal direction) measuring 250 × 50 × 10 mm, maintaining a constant velocity of 5 mm/min. The IB test was performed with three samples from each board measuring 50 × 50 × 10 mm, taken from the outer and inner parts of the board, using a constant velocity of 2 mm/min. The number of samples of each board used in the tests is indicated in Table 3. Before testing, the samples were placed in a JP Selecta refrigerated cabinet (model: Medilow-L, Barcelona, Spain) for 24 h at a temperature of 20 °C and a relative humidity of 65%.

**Figure 2:**
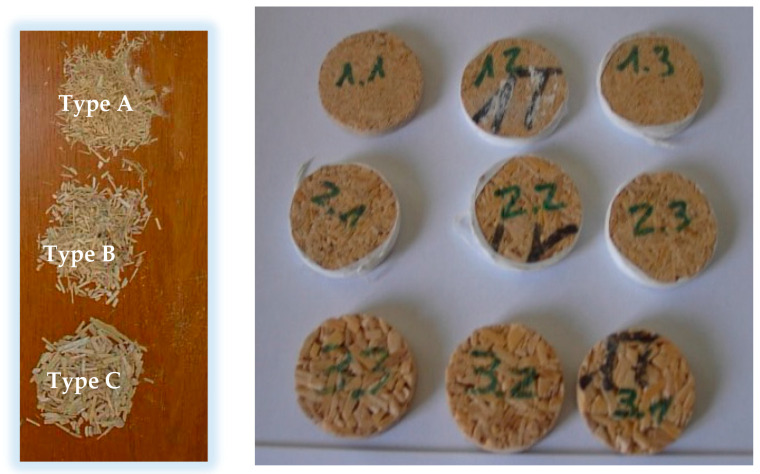
Particles and acoustic test samples.

The method followed to determine the sound absorption coefficient of a material under normal incidence is based on the acoustic impedance tube. This test method uses an impedance tube, two microphone positions and a digital signal processing system [56]. This technique requires a correction procedure prior to testing to minimise differences in amplitude and phase characteristics between the two microphones. The Acupro Spectronics impedance tube (Spectronics C., Lexington, KY, USA) was used for testing, with a frequency range between 50 and 6300 Hz. Three samples of each type of board were used for the acoustic tests. The different samples can be seen in Figure 2.

## 3. Results and Discussion

### 3.1. Scanning Electron Microscopy

**Figure 3:**
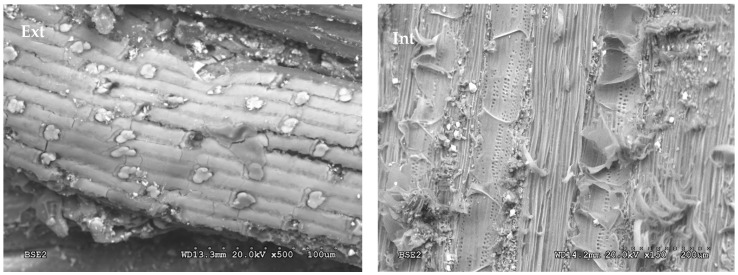
Micrograph of the outer (**ext**) and inner (**int**) surface of the giant reed.

Giant reed is cylindrical and has a hollow interior. Figure 3 shows the micrographs of the outer and inner surfaces of the plant. On the outside, the vascular bundles of compact fibres surrounded by silica phytoliths can be visualised, aligned along the entire outer surface of the reed, giving it a bright, glossy appearance. On the inside, the vascular bundles are less dense and there are small stomata aligned along the surface.

**Figure 4:**
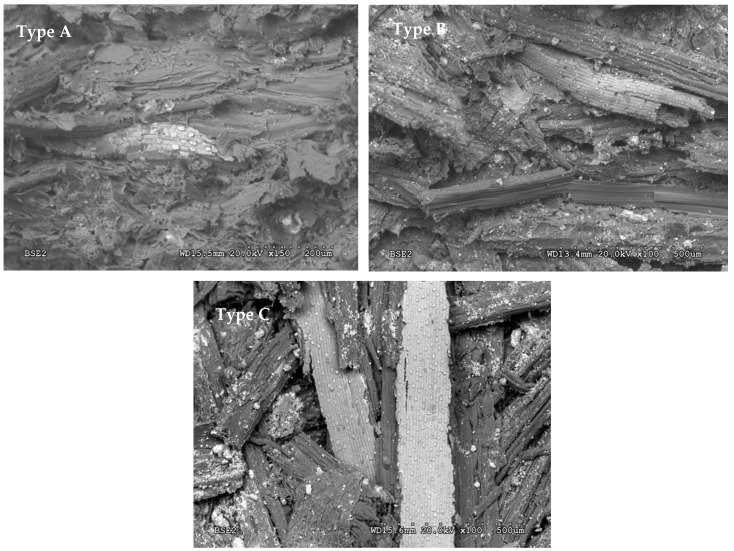
Micrographs of fracture surfaces of **type A**, **B** and **C** particleboards.

Figure 4 shows the micrographs of the fracture surfaces of panels made from type A (0.25 to 1 mm), type B (1 to 2 mm) and type C (2 to 4 mm) particles, respectively, in which it can be observed that the greater the particle size, the greater the porosity.

In the panels with a larger particle size, there is a lot of porosity and it can also be noted that they fracture more easily when the particles are from the outer layers of the reed, where the phytoliths are concentrated, as can be seen clearly in the type C panels in Figure 4. This seems to indicate that boards with larger particle sizes have a lower density and worse mechanical properties, as they are very porous and have a large surface area of phytoliths that prevent better binding of the particles, making adhesion of the urea–formaldehyde resin more difficult. In the boards with small particle sizes, it can be observed that they have a lower porosity, except for the fibres of the vascular bundles, which have not been compressed because they are harder.

Klimek et al. [57] carried out a microscopic evaluation of different particleboards to find structural evidence of differences between the properties found, establishing that particle–particle bonding influences both mechanical performance and thickness swelling.

### 3.2. Physical and Mechanical Properties

For the laboratory tests, the samples for the different tests were previously kept in a controlled atmosphere at a temperature of 20 °C and relative humidity of 65% for 24 h. The mean values obtained in the tests are shown in Table 4.

The density of the boards ranges between 719.6 and 817.8 kg·m^−3^, so they can be considered medium-density boards. Having performed the analysis of variance (Table 5), it can be observed that the density depends on the particle size. The result obtained coincides with our observations in the micrographs of the fracture surface of the manufactured panels: the larger the particle size, the lower the density. When the density of the samples is increased, the amount of particles also increases. Therefore, the excessive use of fibres contributes to the formation of a more compact board, which reduces its capacity to absorb sound waves. This basically means that boards with a lower density have a higher porosity, making them a better acoustic insulating material [15,39].

The average thickness swelling (TS) value of the boards after immersion in water for 24 h is similar, ranging from 19.3% to 20.7%, and does not depend on the particle size used.

The modulus of rupture (MOR) depends on the particle size. The MOR value for type A particles is 17.2 N·mm^−2^, and it decreases with larger particles. The modulus of elasticity (MOE) also depends on the particle size, with values ranging from 1190 to 2300 N·mm^−2^. The internal bonding strength (IB) values do not depend on the particle size and high values are obtained, ranging from 1.0 to 1.3 N·mm^−2^. The result obtained coincides with that achieved in other research [59], in which the authors state that better mechanical properties are obtained with a smaller particle size.

Table 4 compares the results obtained with the values required by the European standards [58] in order to determine the compatibility of uses of boards with a thickness of 6 to 13 mm. In order for boards with type A particles to be classified as Grade P3, a water-repellent product should be added to them to reduce the TS to values below 17%, so they are classed as Grade P2. Type B and C boards would achieve a Grade P1 classification and could be implemented in general applications.

### 3.3. Acoustic Properties

**Figure 5:**
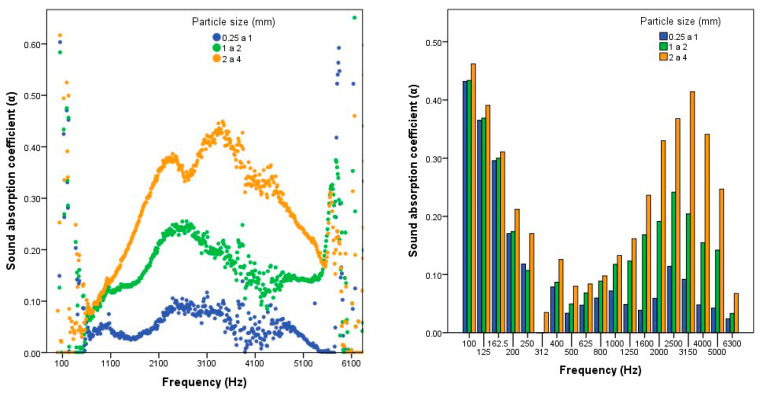
Coefficient α according to the particle size obtained in the test and for standardised octave frequency bands.

Figure 5 shows the average values obtained for the sound absorption coefficient in the tests carried out with the three types of board. As can be observed, there are considerable differences between the boards manufactured according to particle size. Figure 5 shows the average values obtained in the sound absorption test according to particle size for standardised octave frequency bands.

As shown in the graphs corresponding to the sound absorption test, with all three particle sizes, a high value is obtained for very low frequencies (with 50 Hz, the absorption coefficient is 0.7). It then decreases to low values for medium frequencies and increases again for high frequencies. The graphs for the different types of board indicate that there are significant differences between some types of board and others, especially in the 1250 to 5000 Hz bands. In reference to the statistical analysis (Table 3), it can be concluded that there are significant differences with respect to the particle size used, and that the larger the particle size, the greater the sound absorption coefficients. This may be because the boards have different densities, indicating different degrees of porosity, as is also observed in the micrographs. Therefore, boards with a lower density have a greater porosity (or larger particle size), improving the acoustic properties of the boards.

Some studies with other plant fibres have observed similar findings to this work regarding the acoustic performance of the board according to frequency, showing a good performance at low and high frequencies, but a worse performance at medium frequencies [15,23,29,39].

Table 6 shows the sound absorption coefficients based on the standardised centre frequencies of octave bands. The results obtained refer to these frequencies because they are the most commonly used in architectural acoustics and in most of the works and studies consulted. Doing so facilitated a subsequent comparison of the results with the values obtained for other materials of the same density that are commonly used in construction and, therefore, their classification in accordance with the applicable regulations [60].

The results showed that type C boards (2 to 4 mm) are better at absorbing noise than commercial wood and plywood for low and high frequencies, while similar values are obtained for medium frequencies. Type B boards (1 to 2 mm) offer greater sound absorption than wood boards, except at the frequency of 500 Hz. For type A particleboards (0.25 to 1 mm), the values obtained are similar to those of wood boards.

According to the current regulations [60], with sound absorption coefficient values between 0.30 and 0.55, they could be classified as Class D absorbents; with values ranging between 0.15 and 0.25 they could be classified as Class E absorbents, and with values below 0.15 they would not be classified. In general, type C reed particleboards (2 to 4 mm) could be classified as Class D sound absorbing boards, except for medium frequencies ranging from 250 to 1000 Hz.

Other studies with coconut fibre [19] and date palm [23] have also found that an increase in thickness leads to an increase in the sound absorption coefficient, so increasing the thickness of the board would improve the sound absorption properties.

**Figure 6:**
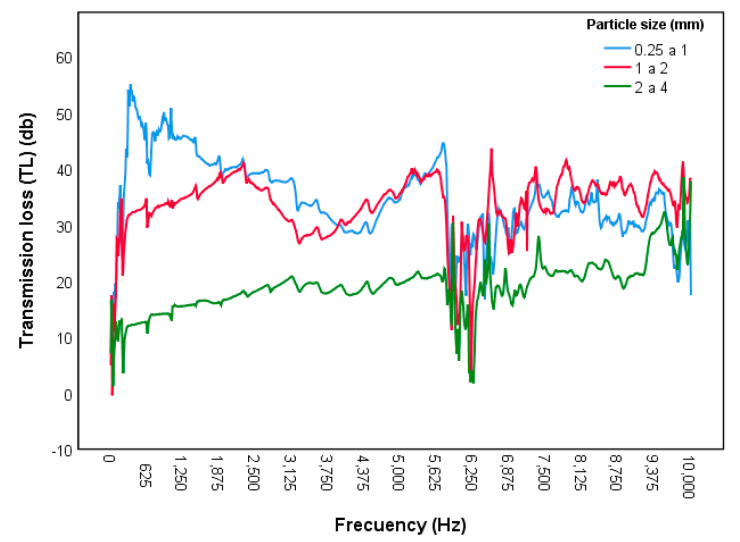
Transmission loss according to particle size.

Sound transmission loss (TL) is a parameter expressed in decibels (dB) that depends on frequency and thickness, and its value indicates how much the energy of the incident sound is attenuated as it passes through a material. Figure 6 shows the average TL values obtained in the test on three samples of each type of board according to the standardised centre frequencies of octave bands.

A larger amount of acoustic energy is lost in boards with a smaller particle size, with the sound energy being lower than the incident energy up to 52 dB at a frequency of 400 Hz. The mean sound transmission loss for boards with particle sizes of 0.25 to 1 mm is 37.5 dB, for particle sizes 1 to 2 mm it is 31.5 dB and for particle sizes 2 to 4 mm it is 15 dB. The sound transmission loss depends on the density of the board and on the particle size. This shows that reed boards can be a good acoustic insulating material, as the sound is attenuated more with smaller particle sizes and the sound absorption coefficient is greater with larger particles.

For wood-derived panels with acoustic properties, MOR values of approximately 1.55 MPa and MOE values of 276 MPa are required [62]. In this regard, we stress that the mechanical properties of all types of boards manufactured exceed these values. Mechanical strength is not generally one of the main requirements in acoustic materials [63], but the boards produced in this work not only have good acoustic performance, but could also be used for applications requiring certain mechanical features in buildings, such as enclosures (vertical and horizontal) and floorings.

The manufacture of industrial particleboards requires more energy consumption than the boards of this study, as commercial wood boards consume a larger amount of energy in the grinding process, as giant red stems are easier to grind than a tree trunk. Finally, in this study, it was not necessary to dry the material after grinding and the pressing temperature and pressure (120 °C, 2.6 MPa) were lower than the temperature and pressure used in the manufacture of industrial particleboards (180 °C, 3.5 MPa).

## 4. Conclusions

The acoustic insulation properties of giant reed boards are conditioned by the spaces between their particles. Using larger particle sizes results in boards with a lower density, achieving a better sound absorption coefficient.

The acoustic results achieved for type C reed boards (2 to 4 mm) are suitable for use as an insulation material for sound absorption. These boards offer better values than commercial wood boards in general and plywood boards used in construction.

The panels manufactured had high sound transmission loss (TL) values, and the thinness of the panels indicated that they had good sound insulation properties. The acoustic properties could be improved by increasing the thickness of the board.

The medium-density boards manufactured have good mechanical properties; the type A particleboards could be classified as Grade P2 and type B and C boards could be classified as Grade P1. All the boards tested had better mechanical properties than those required for boards that are intended for acoustic insulation.

These particleboards could replace traditional raw materials used in construction, contributing to a reduction in the pressure on timber forest resources.

## Figures and Tables

**Table 1:** Chemical composition of *Arundo donax* L. according to different authors.

Components (%)	Shatalov et al. [47]		Pascoal Neto et al. [48]		Caparrós et al. [49]
	Node	Internode	Node	Internode	
Ash	4.77	6.14	3.03	3.80	3.00
*Silicates*	1.31	1.16			
Lignin	20.92	21.31	17.70	19.40	23.00
Holocellulose	61.21	61.41	57.70	62.17	64.50
*α-Cellulose*	29.18	32.93	30.80	34.63	34.80
*Hemicelluloses*	32.03	28.48			

**Table 2:** Types of board manufactured.

Type of Board	Particle Size (mm)	Pressing Pressure (MPa)	Pressing Time (min)	Pressing Temperature (°C)	No. of Boards
A	0.25 to 1	2.6	4	120	8
B	1 to 2				8
C	2 to 4				8

**Table 3:** Dimensions and number of samples of each board used in the tests.

	Density	TS	MOR	MOE	IB	Acoustic
Dimensions (mm)	50 × 50	50 × 50	50 × 200	50 × 200	50 × 50	Ø 30
Number	6	3	3 longit. 3 transv.	3 longit. 3 transv.	3	3

Longitudinal (longit.); transversal (transv.).

**Table 4:** Physical and mechanical properties of giant reed particleboards.

Type of Board	Density (kg·m^−3^)	MOR (N·mm^−2^)	MOE (N·mm^−2^)	IB (N·mm^−2^)	TS 2 h (%)	TS 24 h (%)
A	817.8 (57.1)	17.2 (2.9)	2200 (387)	1.10 (0.4)	13.0 (3.0)	19.3 (7.7)
B	752.6 (53.3)	13.2 (0.6)	1790 (095)	1.30 (0.2)	14.8 (3.7)	19.3 (5.5)
C	719.6 (42.4)	10.5 (1.0)	1190 (189)	1.00 (0.4)	15.8 (7.0)	20.7 (7.3)
Grade P1 [58]		10.5	-	0.28	-	-
Grade P2 [58]		11.0	1800	0.40	-	-
Grade P3 [58]		15.0	2050	0.45	-	17.0
Grade P4 [58]		16.0	2300	0.40	-	19.0

Standard deviation (..).

**Table 5:** ANOVA of the results of the tests.

Factor	Properties	Sum of Squares	d.f.	Half Quadratic	F	Sig.
Type of board	Density (kg/m^3^)	16,726.951	2	8.363.473	3.326	0.047
	MOR (N/mm^2^)	89.879	2	44.940	15.123	0.003
	MOE (N/mm^2^)	1,805,357.706	2	902,678.853	14.897	0.003
	IB (N/mm^2^)	0.226	2	0.113	0.824	0.477
	TS 2 h (%)	105.941	2	52.970	2.181	0.120
	TS 24 h (%)	33.823	2	16.912	0.352	0.704
	Sound absorption (α)	0.434	2	0.217	9.745	0.000
	Transmission loss (dB)	370,834.789	2	185,417.395	2,071.943	0.000

Sound absorption coefficient (α); degrees of freedom (d.f.); Fisher–Snedecor distribution (F); significance (Sig.).

**Table 6:** Sound absorption coefficients (α) according to frequency.

Material	Type of Board	Thickness (mm)	Frequency (Hz)					
			125	250	500	1000	2000	4000
Giant reed panel	A	6.7	0.37 (0.00)	0.12 (0.00)	0.03 (0.01)	0.07 (0.03)	0.06 (0.02)	0.05 (0.01)
	B	6.7	0.37 (0.00)	0.11 (0.03)	0.05 (0.00)	0.12 (0.00)	0.19 (0.08)	0.15 (0.04)
	C	6.7	0.39 (0.04)	0.17 (0.09)	0.08 (0.08)	0.13 (0.06)	0.33 (0.04)	0.34 (0.07)
Wood [61]		25.0	0.19	0.14	0.09	0.06	0.06	0.05
Plywood [61]		9.0	0.28	0.22	0.17	0.09	0.10	0.11
Insulation classes ^(a)^ [60]			D	E	-	-	D	D

Standard deviation (..); classification for type C boards ^(a)^.
